# More work for ASHAs? Exploring the role of whatsapp messaging and community health work in urban slum areas of Varanasi, India

**DOI:** 10.1371/journal.pgph.0005157

**Published:** 2025-10-15

**Authors:** Amelia Jamison, Rohini Ganjoo, Saraniya N. Tharmarajah, Bikash Kumar Panda, Manoj Parida, Shruti Pandey, Madhushree Pandey, Satyanarayan Mohanty, Rajiv N. Rimal

**Affiliations:** 1 Department of Health, Behavior & Society, Johns Hopkins Bloomberg School of Public Health, Baltimore, Maryland, United States of America; 2 Department of Biomedical Laboratory Sciences, George Washington University, Washington, District of Columbia, United States of America; 3 Development Corner (DCOR) Consulting, Bhubaneshwar, Odisha, India; 4 Sarthak, Varanasi, Uttar Pradesh, India; Rural Women’s Social Education Centre, INDIA

## Abstract

India’s community health workers (CHWs) have taken on an onerous set of responsibilities for the welfare of their clients across a variety of health domains, including providing modern contraception for family planning, promoting childhood vaccinations, and enhancing pregnant women’s nutritional outcomes. Governments have also added technology to this mix under the assumption that new digital tools can relieve the work burdens. Through interviews with 22 CHWs, this study found that, besides offering health services for the community, CHWs report an expanding workload that includes several government initiatives. While beneficial in some ways, digital technologies have also introduced new administrative burdens for CHWs, who must manage recordkeeping across incompatible systems. Our findings highlight the complexities of using *mhealth* technology, emphasizing the need for solutions that enhance rather than replace the vital role of CHWs in fostering trust and overcoming barriers to vaccination. While CHWs continue to play a pivotal role in improving vaccine uptake and navigating the complexities of healthcare delivery, their expanding responsibilities and the nuanced challenges of vaccine hesitancy—particularly in urban slum areas—underscore the need for ongoing support and adaptation of technology to effectively bridge the gap between healthcare systems and the communities they serve.

## 1. Introduction

India’s robust Accredited Social Health Activist (ASHA) program has set a world standard for community-oriented healthcare for nearly 20 years [1]. As community health workers (CHWs), AHSAs constitute a female workforce nearly one million strong, providing basic first aid, preventive health services, and facilitating access to both healthcare and government aid [1]. Yet, rapid urbanization, climate change, and population growth have all made maintaining the health of the Indian population an on-going challenge [2,3]. This is particularly true in urban slum areas where limited public health infrastructure and dense living conditions further exacerbate health issues [4]. Indeed, the United Nations estimates that roughly half (49%) of India’s urban population resides in slum areas [5]. In recent years, mobile technology - or *mHealth -* interventions have been positioned as a tool to ease CHW workloads [6,7]. While there is promising evidence that CHW-focused interventions that incorporate *mHealth* tools can be effective at a population level [6], less is known from the perspective of the CHW’s themselves. In this research, we rely on interviews with CHW’s working in India’s urban slum areas to explore the potential for a WhatsApp-based *mHealth* intervention designed to promote childhood immunization.

### 1.1 Role of community health workers

The Indian healthcare system relies heavily on CHWs, as frontline workers operating on the periphery of the formal system, to deliver primary healthcare to diverse communities [8]. The Ministry of Health and Family Welfare introduced the ASHA program in 2005, first in rural areas, and then later expanded into urban areas [1]. While AHSAs are the center of this model, they are supported by other CHW roles, including specialized Accredited Nurse Midwives (ANMs) and community health center workers, known as Anganwadi Workers (AWWs). Each ASHA must meet certain criteria. She must be: 1) a woman; 2) *from* the community she serves; 3) married, widowed, or divorced; 4) between ages of 25–45; and 5) formally educated [9]. Recent studies have underscored the vital role of CHWs as frontline workers in India, instrumental in gains in maternal and child health, routine immunization, nutrition support, and overall health and wellbeing [10,11,12,13].

### 1.2 Urban health nutrition days

The Indian government actively supports social policies through the public provision of universal programs in several domains, including public education, social security, public amenities, and public health [14]. This programming has been upheld by universalistic principles that ensure services are open to the entire population on a non-discriminatory basis. Among these universal initiatives, Village Health Nutrition Days (VHNDs) and Urban Health Nutrition Days (UHNDs) were created as platforms to offer preventive services for the entire population, while specifically focusing on vulnerable communities [9]. Comprising four components, UHND programming focuses on 1) health, 2) nutrition, 3) early childhood development, and 4) sanitation services. Objectives of UHNDs include improving accessibility of services, creating awareness about various governmental programs, and serving as a platform to connect individuals to these four domains at the community level. UHNDs are typically held once a month at an Anganwadi Centre, with frontline services provided through ASHAs and AWWs. As part of this design, all health services, including antenatal care, immunization, nutrition, and family planning, are provided at the same location and the same date, with no cost to patients [15]. Within the immunization activities, programming focuses on providing routine vaccinations to eligible children and catch-up immunization to those who have missed doses [15].

### 1.3 Immunization disparities in India

Childhood immunization rates can serve as an indicator for the quality of a nation’s health care system, as successful immunization requires not only effective systems of healthcare delivery, but also high levels of confidence across the population [16]. Since 1985, India’s Universal Immunization Programme (UIP) has provided federally funded vaccines to more than 25 million children annually [17,18]. The *Mission Indradhanush* government initiative was launched in 2014 to increase childhood vaccination rates to 90% for 12 childhood vaccines, significantly boosting vaccination rates across India, from 62% in 2015/16–70% in 2019/21 [19]. The COVID-19 pandemic interrupted this trajectory; the WHO estimates India experienced the greatest disruptions in delivery of routine childhood immunizations [20]. These losses were starkest in regions with the lowest pre-pandemic healthcare capacity, such as urban slums [21]. It remains to be seen whether these declines reflect a short-term disruption in care, or if they mark a new trend in vaccine hesitancy [22]. In either condition, these declines also suggest ongoing challenges in the overall healthcare infrastructure.

There are also significant immunization disparities that persist nationally, with slum areas experiencing lower than average uptake rates [23]. Life in slum areas is marked by unstable employment, low education, minority status, and housing insecurity, compounding existing childhood vaccination challenges [24,25]. People living in slum areas may also have pressing domestic and economic concerns, making vaccination a lesser priority [26]. Post-COVID declines in childhood immunization are especially worrisome in these environments, exacerbating existing issues of limited vaccine access and infectious disease outbreaks [27,22].

### 1.4 mHealth

The World Health Organization recognized *mHealth* (sometimes under the broader umbrella term of *eHealth*), defined as “the cost-effective and secure use of information and communication technologies in support of health and health-related fields” [28]. However, challenges remain in using *mHealth* technologies for intervention dissemination and evaluation [28]. *mHealth* interventions have been heralded as a solution for building networks for community health work at scale, particularly in low-and-middle-income countries (LMICs; [7]). Literature and scoping reviews of the evidence have tempered this early enthusiasm, often due to the lack of formal evaluation on many projects [6,7]. More recently, a combination of increased smartphone penetration in LMICs and advances in technology (e.g., AI-driven chatbots) have reinvigorated interest in *mHealth*.

While studies increasingly measure the impact of *mHealth* applications on the underlying health outcome of interest, we were curious to learn how these applications impact community health workers directly. We conducted qualitative interviews with CHWs working in the slum areas of Varanasi as part of our larger research project to develop an *mHealth* application to promote childhood immunization, later named the *Happy Baby Programme*. This allowed us to explore the CHW perspective on both *mHealth* applications and CHW roles more broadly. In this analysis, we explore two interrelated research questions: first, how might *mHealth* applications impact CHW roles related to vaccination? And second, how do *mHealth* applications fit into CHW workloads more broadly?

## 2. Methods

### 2.1 Setting & context

Our work was based in Varanasi, a city in the Indian state of Uttar Pradesh. Varanasi has a Hindu majority (70%) and a Muslim minority (29%) [29]. The city has undergone rapid urbanization, including a rise in slum settlements. The United Nations considers a slum dwelling as a type of insecure housing where multiple individuals reside without access to basic services [30]. The Indian government recognizes “notified slums,” a legal status that provides greater security against slum clearance efforts and access to more government resources [4]. In Varanasi there are 210 officially “notified” slum areas. Of these, we selected six of these for our trial, which we then numbered 1–6, to maintain anonymity.

### 2.2 Project design

We conducted interviews with frontline workers as part of a larger project that included a behavior change intervention trial (reg [blinded]) designed to test two different WhatsApp-based messaging campaigns against a control group. Preliminary findings from the behavior change study are published elsewhere ([blinded]). The project was also registered with the U.S. National Libraries of Medicine, with a complete overview available at clinicaltrial.gov. The analyses described in this paper focuses on qualitative data collected to understand the larger health context in which mobile messaging technologies would operate.

### 2.3 Study design & sample

We conducted a qualitative study of CHWs working in the six slum areas designated for our *mHealth* intervention. Our local partners at [blinded] had established relationships with Urban Primary Health Centers and Anganwadi Centers in Varanasi. These established relationships allowed us to directly recruit CHWs to participate in-person interviews on location at health centers. We also contacted the medical officers (known as MOICs in India) in charge of each district for interviews. All CHWs at affiliated sites were eligible for participation. Research coordinators engaged in purposive sampling to intentionally select participants representing both a range of CHW roles across site locations. Table 1 describes the relationship between each of these roles (MOIC, ANM, ASHA, AWW) and the hierarchical organization of the Urban Primary Health Centers and Anganwadi Centers.

**Table 1:** Role of community health workers.

Community Health Worker	Role	Responsibilities
Medical Officer in Charge (MOIC)	Head of the Urban Primary Health Centers	Oversee all preventative, curative services, health education and promotion and personnel. Each Urban Primary Health Center serves a slum area of a population between 30,000–75,000 people.
Accredited Nurse Midwives (ANM)	Serve under the MOIC 3-5 ANM per Urban Primary Health Center	Provide services at UHNDs including: • pre/post-natal care • routine immunization • family planning services • growth monitoring and nutrition counselling • minor medical treatments • provision of medicine • referrals and follow-up.
Accredited Social Health Activists (ASHA)	Work with ANM 4-5 ASHAs per ANM	Support ANMs: • conduct home visits • accompany patients during health care visits Responsible for roughly 1,000–2,500 people
Anganwadi worker (AWW)	Based at the local Anganwadi Centers	Deliver essential services to Anganwadi Center patients, including: • supplementary nutrition • routine immunization • routine check-ups • referral services • pre-school education • health and nutrition education.

### 2.4. Data collection & analysis

Our analysis plan was based on constructivist grounded theory [31]. Like traditional grounded theory, a constructivist grounded theory relies on an inductive and iterative approach to qualitative analysis, rooted in direct engagement with the accounts from participants. However, by embracing a constructivist bent, we reject the idea of theory development as approximating an underlying “truth” and acknowledge that our own theory generation is an act of construction [32]. This was important for our approach, as an interdisciplinary, multi-ethnic, international research team. Rather than render our roles in this analysis invisible, we wanted to “ground” our analysis to reflect the voice and experience of the participants, but simultaneously recognize our own team’s subjectivity as an active presence shaping our interpretations [32].

As such, our positionality as researchers is necessary context [Naidu et al. 2024]. Our team includes members reflecting a range of professional and lived experiences. The India-based teams included both a local research team led by a senior PI (blinded) and a data collection team supervised by (blinded). The data collection team consisted entirely of Indian women trained as interviewers, and all were native-Hindi speakers and based in the Varanasi-area. The US-based data analysis team consisted of ethnically diverse researchers with qualitative, social science, and health backgrounds working in research universities (blinded). This team included an individual identifying as White as well as first-generation and second-generation immigrants from Southeast Asia (India and Nepal). As researchers aware of the deep power imbalance in the traditional practice of global health research, the team sought opportunities to center local expertise throughout all stages of research design, implementation, analysis, and dissemination.

The data collection team worked in pairs (one interviewer and one notetaker) to conduct face-to-face interviews. Interviews took place either on-site at Anganwadi Centers or in the field, depending on the participants’ availability and preference. Interviews were conducted in Hindi, recorded for transcription and translation and averaged between 45–60 minutes. All data collectors received both comprehensive training on qualitative research and project-specific training on the interview protocol prior to conducting the interviews. We intentionally chose all-female interview teams, to help build rapport with the all-female CHW teams. Similarly, we note that both interviewers and CHW interviewees were similar in other ways, including in education levels and in religious backgrounds [all were Hindu].

Members of both US-based and India-based research teams collaborated on interview guides, drawing on themes found in exploratory qualitative data collection, including sources of vaccine hesitancy, barriers to technology use, and workplace roles. The interview guides were written in English, then translated into Hindi by team members proficient in both languages. This translation process was designed to maintain both cultural and linguistic nuances and to ensure that the questions resonated with the local context.

Accordingly, our data analysis was an iterative process. First, two team members (AMJ & RG) read through a sample of transcripts, marking them up by hand (underlying, labeling, and inserting notes) to identify key concepts and ideas to develop an initial codebook of high-level codes [examples included: “role,” “vaccines,” “community”] (Table 2). This initial codebook of high-level themes was then approved by the entire qualitative research team, including the data collection team. At this point, a single coder (AMJ) began annotating transcripts using qualitative analysis software (MaxQDA24). This coding included both high-level codes, as well as more refined axial subcodes (examples under the code “role” include; “role: boundaries”; “role: responsibilities”; “role: qualifications”; “role: community expectations”; etc.) (Table 2).

**Table 2:** Qualitative codebook.

High-level Codes	Subcodes
Community	Gender roles; Muslim community; Tribal community
Food/Nutrition/Support	Rations; Election/Census; Schemes; Family Planning
Role	Community Attitudes; Travel; Workload; Job; Boundaries; Credentials/Experience; Responsibilities
Technology	Benefits; Easy Communication; Barriers; Use of Apps
Vaccines	Barriers; Refusal; Demand; Hesitancy; Rumors; VHSNDs

Throughout the coding process, our team engaged in collaborative memoing, a process that involved writing up thoughts, impressions, and emerging ideas, heavily illustrated with direct quotes [33]. Memos became a collaborative space, as the team weighed in to clarify, affirm, question, reject, or deepen emergent ideas. In the process of coming together as a team, these memos also allowed both the *emic* (or “insider”) perspective and the *etic* (or “outsider”) perspectives to emerge [Galperin et al. 2022] [34] . This made it possible to describe CHW work within the local context as experienced by data collectors, while also exploring more global themes across CHW work and across academic literature. Two memos (one on vaccine hesitancy and a second on the relationship between CHW work and technology) serve as the foundation for this manuscript. To enhance methodological rigor, as a form of member checking, the US-based team drafted a presentation of emerging qualitative findings and presented them to the India-based team at an in-person summit to understand which aspects resonated and which ones did not.

### 2.6 Ethical considerations

We obtained ethical approval from the US-based review board at the Johns Hopkins Bloomberg School of Public of Health (IRB23660) where it was deemed to be exempt. The overall study also received IRB approval from Sigma Consulting in Delhi, India (10022/IRB/23–24). Recruitment for this study began on 29/12/2023 and ended on 20/01/2024. Verbal consent was obtained from participants, which was recorded by the interviewer. Due to the minimal risk associated with participation in the study, the IRB waived written consent, as in the event of a data breach signed consent forms would actually add to the risk to confidentiality of participants.

## 3. Results

We spoke with 22 health workers across different roles (Table 3). Demographically, all participants identified as female and were between the ages of 20 and 56. Participants had between 1–8 years of experienced in their roles (median years of experience was between 2 and 3 years), had some form of higher education and were Hindu. These last two factors are particularly noteworthy, as one of the strengths of the CHW role is that these workers often share the lived experiences of the communities they serve, and we recognize that our CHW population may be meaningfully different. Representative data from the community show, for example, that approximately a quarter of caregivers in the community had completed a secondary level education and 27% were of religions other than Hindu [Rimal et al., 2024] [35] . Given that some form of higher education is a prerequisite for a CHW role, the differences in education level are not surprising. In contrast, differences in religious background may be more salient. Our sampling procedure did not take religious background into account; it was only after it emerged as a significant theme in analysis that we recognized the need to determine the religious background of all participants. Official documentation is limited, but accounts suggest that ASHA workers in this region may be disproportionately Hindu, even those that serve in majority-Muslim districts.

**Table 3:** Participant demographics.

	Total
Role	
AWW ASHA ANM/GNM MOIC	8 6 5 3
Gender	
Female	22
Age (yrs)	
20-29 30-39 40-49 50+	1 12 6 3
Experience (yrs)	
<5 5-10 >10 Unknown	5 7 8 2
Slum District	
S1 S2 S3 S4 S5 S6	3 5 4 4 3 3

### 3.1 CHW role

In addition to delineating the technical responsibilities of each CHW role, we wanted to understand how CHWs themselves understood their own positions. Many of the CHW’s presented themselves in terms of the services they provided, often listing tasks, duties, and obligations (e.g., “*Our responsibilities include participating in UHNDs and looking after pregnant mothers*” (S5-ASHA) or *“My work is to conduct vaccination drives and give advice to pregnant women”* (S4-ANM)). For the majority of CHWs, job descriptions were based on the population being served, typically pregnant women and children. Reflective of their broad responsibilities, they described a wide range of services geared towards these groups, including pre/postnatal care, nutrition, family planning, health education, and even schooling. When explicitly mentioned, immunization was tied to the broader goal of keeping these populations healthy. For instance, one ASHA described her priorities:

*See, my primary focus is family planning. It is our responsibility to work on the maternal mortality, which is increasing these days, or the infant mortality, which is increasing. That is why we work can be family planning... our main responsibility is to take the pregnant women to the health care center for their delivery and get small children vaccination on time* (S5-ASHA).

At the same time, CHWs described growing workloads that extend beyond direct health services. This included administrative roles in government initiatives, support for census and elections, and distribution of aid. Yet, given their low position in the healthcare hierarchy, they felt obligated to take on this new work, even if they were already feeling overworked. In one exchange, one CHW described her work burden, but then reassured the interviewer that, “*Whatever work we’re assigned, we do it from our heart with full responsibilities...*” (S1-ASHA). One even hinted at her displeasure at having to complete our interview as her other work obligations continued to accrue.

### 3.2 Vaccine hesitancy

CHWs described relatively high confidence in immunization and few challenges with vaccine hesitancy. CHW’s used language that connects themselves to these successes. For instance, “*In our area*, *most people take the vaccines, only a few are there who don’t take*...” (S3-AWW) and “*In our field*, *it’s not like we need to convince them. We just inform them and they come*” (S2-ASHA). Some CHWs described a trend of increasing demand for vaccines. Perhaps, in the past, community members needed to be persuaded to receive vaccines, but now CHWs described patients who actively seek out vaccines. The motivation was coming from patients, as one ASHA explains; *“No, no women are not afraid now. Women are aware now; I should be healthy. If a mother will be aware herself, then the child will be healthy, and now the mothers are aware that, ‘My child should get a vaccine on time’*...” (S3-ASHA). Importantly, the CHWs suggested a connection between UHND attendance and both vaccine awareness and acceptance.

Yet, all CHWs acknowledged that vaccine hesitancy existed and posed a challenge. This was typically characterized by parental concerns about vaccine side effects, particularly acute, short-term side effects such as fever or swelling at the injection site. Nearly every CHW we interviewed could describe a time where a concerned parent had expressed fears related to side effects. For instance, this description from an AWW; *“And those who do not want to get vaccinated refuse on their face... some people get scared because there are some children who get fever immediately after the vaccination...*” (S5-AWW). At the same time, the CHWs were confident that they had the training to respond in such scenarios and often followed-up with the medical advice they would recommend for parents *“give the child medicine and massage with ice...*” (S2-ASHA) and “*give medicines and syrups”* (S3-MOIC). Some CHWs suggested that the presence of side effects was proof that the “*medicine is working*” (S6-ANM). Importantly, these recollections suggest the CHW-patient exchange was primarily focused on filling information deficits related to common vaccine side effects, not that community members were expressing deeper concerns about either the vaccines themselves or the practice of immunization more generally. By providing reassurance and practical advice, CHWs not only bridged the gap in knowledge but also demonstrated their commitment to the well-being of community members, thus nurturing trust. Indeed, most CHWs could point to these exchanges as positive moments and opportunities to build trust:

*They say that their children get a fever after getting a vaccination. Then you go. Because after some days of vaccination, we again visit their houses to know about the health of the children. They get satisfied as we visit them before and after vaccination; as a result, people build trust in us* (S1-ASHA).

While CHWs described low hesitancy among those they encountered at UHNDs, they indicated – through omission – that these descriptions would not apply to those who *do not attend* the UHNDs. All the CHWs acknowledged the many barriers that may keep community members from attending a UHND. Foremost among them was the extreme poverty of the region and that families were busy laboring to make ends meet. CHWs described this challenge not only as it impacted attendance at UHND events, “*The biggest challenge for us is that people here are always busy with work, which is why they do not have time, so no one comes on time for vaccination whenever we want to ask”* (S5-ASHA). But also, for the way it influenced how parents understand the severity of vaccine side effects;

*Some women who work outside, like sweeping or cleaning, may not show interest because the children may get a fever for two or three days... If the child gets sick it disturbs them, and their work remains pending. That’s why they delay it, but eventually, they get it done* (S6-ASHA).

These challenges were further exacerbated by bad weather, poor travel conditions, or external events like elections.

### 3.3 Entanglements

CHWs described an interesting paradox: community-level vaccination rates were low and competing demands made immunizations rank low among household priorities, *yet* individual levels of vaccine confidence were quite high and were reinforced through interpersonal relationships with CHWs. Vaccination improvements thus appeared to be more about facilitating access than about persuading patients that vaccines are needed. Once a patient attended a UHND, for example, CHW’s were confident in their ability to persuade and administer vaccines to most community members. This became clearer when understanding the full spectrum of services – both medical and non-medical—that were provided during UHNDs. Community members relied on CHWs not just for health care, but also as a conduit to the resources needed for daily life.

To illustrate this connection between vaccines and other services, it is helpful to return to the words of the CHWs as they described all the UHND services, including the receipt of rations, financial incentives, and government aid. The connection between vaccines and food was especially strong. An AWW described how she linked vaccines to rations as an incentive, “*We tell those who feel like coming, ‘Look, this time if you do not come for vaccination, then I will not give you the ration that comes from the government. I will not give it to you...”* even describing how she used toffee and biscuits to *“lure”* children to receive vaccines (S5-AWW). Community members also made this association, as evidenced by the complaints lodged to CHWs when vaccination was not accompanied by rations. As an ASHA explained:

*They complain to us about not getting ration after getting vaccination. Then I make them understand, “Didi it’s not my work to avail ration for you – I’m from the health department...” I told them the usefulness of vaccination, that it’s given for the children’s protection so you should have it. “All the expenses are taken over by the Government - you don’t have to spend a single penny. Take the wholesome benefit of it.” They didn’t have complete knowledge about it. They were saying “Why would we take the vaccine when the Anganwadi is not give us rations properly?”* (S1-ASHA).

CHWs were also actively engaged in other government programs and initiatives. A current priority for ASHAs is registration into the *Ayushman Project,* an initiative to increase access to free health care treatments and services for the lowest income families. Another set of schemes focused on direct financial assistance for pregnant women and new mothers. For instance, the *Mantri Matru Bandana* Program was designed to provide monetary assistance for pregnant women – particularly those who give birth to more than one female child. Yet, as some ASHA’s described, the distribution of funds is staggered and tied to vaccination:

*We work for Mantri Vandana, in which there is a scheme of giving 5000 rupees on the first delivery and 6000 rupees for the second delivery. This scheme is linked to when the mother is pregnant, so at the time of registration, 100 rupees are given; in the first ANC checkup, 2000 rupees are given; and 3000 rupees are given after the delivery. A person can also avail of its benefits after the immunization of their baby with Penta 3* (S4-ASHA).

This connection between financial assistance and vaccines was sometimes made more explicit, “.*.. when the child is 3.5 months old, he takes the vaccine for three and a half months, the woman gets*₹*2000*” (S5-ASHA) and “*Pregnant ladies get this in three installments. When she gets the first vaccine injection, at that time *₹*2000, during the second TT injection *₹*1000, and after delivery when the baby completes its 3*^*rd*^ *month vaccine, at that time she gets *₹*2000*” (S6-ASHA).

To further illustrate this connection between CHWs, interpersonal trust, and government benefits, it is worth looking at the instances where this system falls apart –in the few named instances of vaccine refusal. Only a handful of CHWs described scenarios beyond hesitancy, where community members actively refused vaccination. Interestingly, these instances were prefaced by the CHW distancing themselves from the hesitant party (“*See, firstly* ***they*** *don’t take the vaccine*...” (S1-AWW)). The clearest distinction was across religious and/or tribal lines: *“We face problems with Muslim areas during our field work. They don’t get vaccinated*” (S3-GNM) and “*People who are informed get the vaccines, but there is an Adivasi [a local tribal group] area nearby where they still refuse to hear*” (S1- ANM). Another tack was for CHWs to associate refusal with a lack of education or understanding. However, unlike the hesitant parents who were described as receptive to new information, CHWs characterized the parents who refuse as “*uneducated*” (S3-AWW), “*arrogant”* (S3-ANM), and “*rigid in their thought process”* (S2-AWW). This contrasts with what our quantitative data showed: an absence of a relationship between religion and vaccination and between education and vaccination (XXXX et al. 2024; masked for blind review).

Additionally, rejection of vaccines was also connected to rejection or refusal of other services, including rejection of family planning services for some patients. Some CHWs described how rumors linking vaccines to infertility were common in the communities they served:

*Yes, they’re Muslim. They tell that if they would get the vaccine, they won’t have a child. If they take the polio vaccine, they won’t get a child. They have a very negative mindset. Some openly talk about it and some keep it inside them* (S1-AWW).

Having larger families was also implicitly connected to ideas about who was responsible for them – and some CHWs seemed to suggest that rejecting norms around family size could be accompanied by a broader rejection of government aid services broadly. For instance, an ASHA working in a Muslim region described; “*They have different mindsets. They say, ‘It’s my child, I will take care of it and I am not asking the government to feed my children”* (S2-ASHA). Unlike the inclusive language used to describe vaccine uptake in the community, CHWs were clearly differentiating themselves from the “they” of non-compliant.

However, some CHWs recognized that norms may be shifting, and that, with work and perseverance, change would be possible. For instance:

W*hen I joined, I visited the Muslim slum area. The kids weren’t vaccinated. Kids as young as a few months to kids aged 5 or 6 just weren’t vaccinated. With the help of the Anganwadi, I was able to help vaccinate them. It was very difficult, but it feels good to see these changes. Now, people recognize us and are cooperative (S1-ANM).*

It is unclear whether this change permits acceptance of just vaccines, or if it necessitates embracing the complete package of health care services or the broader social values that they represent. It may be telling that one MOIC used the phrase “*they get converted*” (S4-MOIC) to describe the slow process of earning acceptance from Muslim communities. There was little talk on how the CHWs themselves could change their approach.

To summarize, we found it important to contextualize vaccine acceptance within the broader exchanges that took place between a CHW and the community she served. Vaccine decisions are entangled with decisions about the full array of both health and social services offered at UHNDs (as well as the social and cultural values these services represent). As CHWs serve as a connection between the community and city/state institutions, the CHW-patient relationship is often the foundation for this exchange. Further, CHW-patient relationships are also *social* relationships, the patients recognized as part of the “in-group” in the social exchange tend to receive the full package of services, while those who are othered as the “out-group” may not.

### 3.4 The role of mHealth

When considering this broader system of exchange, we wanted to understand the specific role played by *mHealth* interventions. In what ways could mobile phones, messaging applications, and automated reminders impact the daily work of CHWs? Is it possible to use messaging technologies to improve vaccine uptake without placing additional burdens on CHWs?

For this specific perspective, we considered how CHWs described their role in the community. When narrating their own work histories, most CHWs described the initial challenges that they faced to get established as they entered their fields. As they described, the communities had no sense of the CHW role and were not very willing to open their doors to receive services. As an ASHA explained:

*It has been easier [now] because earlier, when I came to work here, people did not know us; people did not even talk to us, but what is happening now is that people know me and talk to me. Nowadays whenever someone has a problem, he’ll come to me, talk to me, and asks for solutions to his problems* (S5-ASHA).

This is particularly true for those with the longest experience (10 + years). These women contrasted the harshness (e.g., “*people were not talking to us properly..*.” (S2-ANM)) and derision (e.g., “*people used to ridicule like ‘Who is she?’ “Why is she doing that?’”* (S3-AWW)) they received at the start of their placements to the trusted role they now occupied. Only through time and repeated interactions were CHWs able to build rapport, establish relationships, and earn the trust of the community. This was summarized by an ASHA:

*Earlier, we didn’t get recognition in society; women used to refuse to go along with us anywhere; they did not have faith in us when we used to tell them about various schemes, and if ever we took them to the hospital, they used to abuse us for that... Now, they trust us, they listen to what we say, and they show their interest...* (S4-ASHA).

These sentiments aligned with our earlier discussion on the ways CHWs defined their roles, with the relationship itself as the primary focus and the specific tasks as secondary.

In this context, technology can serve as a bridge, helping to maintain connections between CHWs and a preoccupied public. We heard from CHWs that messaging platforms helped ease communication with patients, reduced the need to travel from home-to-home, opening time for CHWs to engage in other tasks. Most significantly, messaging allowed CHWs to reset patient expectations and streamline visits to UHNDs. By sending messages in advance, CHWs could educate community members, who would then arrive ready to avail themselves of vaccination services. As one AWW summarized, “*Before [the app] the situation was good, but we had to inform people by visiting house to house. Now, everyone is aware of us beforehand*” (S3-AWW). Yet, messaging apps did not eliminate all communication barriers, as CHWs described; they sometimes created new ones. Low literacy created challenges in the slum communities, as one ANM explained that ensuring that messages were received and understood was critical, “*If they are unable to read and write, how will they [know to] come?”* (S2-ANM). Technological literacy was another challenge, as phone ownership does not automatically confer mastery of technology. This work also fell to the CHW, as one ASHA explained, “*There are many people who do not even know how to use the phone; they do not understand what to do or what not to do, so we, Asha Didi, help them a lot. We try to solve whatever problems they have*” (S5-ASHA). The CHWs hinted at an emerging disparity between the “educated” and “uneducated” where those who had the capacity could act based on app-driven messages alone, but those who did not have the capacity required additional help from CHWs. For instance, “*Okay, those who are educated come here directly, but those who are not educated – it is not written; but we have to go to their house and tell them about the programme and then they come”* (S5 -AWW).

The CHWs described the administrative burdens that came with relying on new technology. Not only were CHWs responsible for learning new systems, but they also complained about how the lack of compatibility between systems meant they were often presented with additional work through recordkeeping. For instance, one ANM described, “...*due to online work, the work has doubled. We’re to make hard copy and soft copy both...*” (S3-GNM) a sentiment echoed by an ANM who described a system of double recordkeeping, “*We have to upload the information twice; once in the phone, then in the register*” (S2-ANM). It was not clear if the amount of data being collected and stored had increased as well, as new vaccines and new initiatives were introduced. For instance, an AWW described all the things she tracked on a single Anganwadi application:

*BLO [political information], entering details of pregnant women, separate details of children in between 12 months to 3 years, from 3 years to 6 years, lactating mothers, then we enter THR (take home rations) when we distribute nutrition-based food. We enter THR every 25 days. Then we enter vaccination details; vaccine of 9 months, BCG, penta, whatever... Then we enter details of yoga diwas, Annaprashan, Godbharai. It means we enter every detail that we’re working on in Anganwadi, and we do it on that particular day…* (S1-AWW).

The CHWs described a work situation where they faced increased responsibilities in the number of tasks that they performed – in both medical and non-medical services. New technologies including mobile messaging have simultaneously eased some burdens (such as reduced travel, increased ease of communication) while introducing some new ones (including administrative burdens, increased monitoring, and translation work).

## 4. Discussion

In this qualitative exploration of CHW perspectives, we gained insight into two interrelated issues: the role of technology in CHW work and the role of the CHWs in the community. Taken together, this project provided tremendous insight into both the potential capabilities and pitfalls of embracing *mHealth* applications for CHW-driven health interventions.

Technology served to both ease some specific forms of labor (travel, communication), while also increasing others (recordkeeping) and even creating new forms of labor (translation, tech support). In this regard, we saw parallels to Cowan’s influential 1983 work “More Work for Mother” that tracks the historical trajectory of technological development and domestic labor. Cowan (1983) outlines the ways new tools (e.g., washing machines, refrigerators, vacuum cleaners, etc.) “simplify” labor by creating novel forms of labor, the responsibility for which increasingly falls on the women in a household. Importantly, she situated individual work tasks within larger work processes, and each new tool within broader technological systems [Cowan, 1983 [36] ]. In this way, while the uses of new technological tools reflects an individual’s choices, it also must be understood within the broader context of societal pressures outside of the individual’s control [Cowan, 1983]. While *mHealth* applications are embraced, utilized, and valued differently by each CHW, their collective status at the bottom of the medical hierarchy makes it difficult to understand if the adoption of new technologies is truly a choice. As workloads and caseloads continue to grow, the number of hours CHWs can dedicate to work remains constant. An efficient, integrated, and well-supported *mHealth* application could facilitate rapid two-way communication, reduce time-consuming travel, and even automate routine communications (such as reminders for UHNDs). This line of thought implies the need to provide a back-stopping support system when new technologies are introduced, one whose need may diminish over time as the technology gets more widely adopted and understood.

Enthusiasm for new and emerging technologies – such as AI - have opened the possibility of additional avenues for *mHealth* applications. Yet, we found that the CHW system functioned based on the established relationships between the CHW and her community. Could there be unintentional impacts on trust that occur when aspects of interpersonal relationships are mediated through mobile technology? In the related field of telehealth, research has shown mixed effects; when done well, telehealth interventions can enhance public confidence and trust, but other studies have shown neutral or even negative impacts [37,38]. For already-established relationships, *mHealth* interventions may serve a different purpose than those designed to supplant an existing relationship or foster a new relationship from scratch. Research on trust and *mHealth* has tended to focus on issues like data security and privacy [Schnall et al. 2017] [39] , largely overlooking the impact of *mHealth* on interpersonal relationships [40]. Future research is needed to explore *mHealth* potential along these lines, particularly focused on aspects of interpersonal relationships, including trust.

On the issue of childhood immunization, we found CHWs described how the influence they had on patient vaccine decision-making was made possible only through community trust. Existing research has shown that CHW programs work best when the workers themselves are “embedded” within communities [41]. In our study, we observed that when trust levels were high (such as when working with an “in-group”), patients tended to accept the total program of services: vaccines, nutrition, financial aid, and family planning advice. Yet, for those few “outsiders,” rejection of any one of these services sometimes meant a rejection of the entire program. That these outsiders frequently occupied minority status further complicated and compounded this issue. Other scholars have found similar disparities in healthcare utilization by caste, socioeconomic status, etc. – even with narrowly focusing on ASHA-provided services [42]. These findings all suggest that community trust must be considered as an integral component in the development and design of *mHealth* interventions.

Is it possible that *mHealth* interventions could accelerate differences in trust? We hypothesize that for those who already trust a CHW and the system she represents, the added efficiency of online communication can boost engagement and health outcomes. But for those who do not trust the CHW or the system, could *mHealth* interventions contribute to communication barriers becoming entrenched? The CHWs here described the process of rapport building as one that was constructed around many face-to-face contacts. *mHealth* applications designed to reduce face-to-face contact may also unintentionally reduce opportunities to establish relationships. These effects may be further impacted by social divides, such as those based on religion, caste, and/or tribal affiliation. Here we note that while CHWs participants were from the communities they served, none of the interviewed CHWS were Muslim, which likely contributed to levels of trust (or lack thereof) between members of the community and the CHWs. The extent to which including concordance between a CHW and her community in terms of religious beliefs, caste status, or tribal background directly impacts trust relationships and indirectly impacts *mHealth* outcomes toutcomes remains to be seen, This was a limitation of this work that could be addressed in future work that intentionally incorporates these differences into the study design The very premise of *mHealth* depends on the buy-in from users, who then take on additional responsibilities through the application, typically of learning, monitoring, and reporting their own health, shifting the work of professionals onto the individual users [43]. What roles can CHWs serve in this transition?

Finally, despite the significant role of CHWs in the Indian healthcare system, this work hints at the ways ASHA work can be undervalued by both the Indian government and by the communities being served. Pre-pandemic many CHWs reported being overworked and underpaid, conditions further exacerbated by COVID-19 [44]. We saw that these stressors were compounded by the hierarchical nature of the Indian healthcare system, with CHWs occupying among the lowest paying and least respected positions [45]. As both CHW workloads and stressors increase, recent scholarship describes Indian CHWs in untenable situations [46,44,45]. Indeed, there is a growing recognition that worldwide, CHWs occupy social positions that leave them vulnerable to marginalization in the healthcare workforce [8]. With this understanding, we believe our work argues for a more inclusive process of incorporating CHWs in all aspects of *mHealth* design, development, and implementation. We also observed the need to acknowledge that the “timesaving” benefits of *mHealth* may in fact represent largely invisible work of CHWs.

There are several important limitations to this work. First, as we have indicated, the CHWs we interviewed were all Hindu with no one to offer the perspective of Muslim CHWs. We have described how this has impacted both our research process and our findings throughout the work. Second, given the close relationship between our data collection team and the local health centers, interviews were conducted with participants who likely knew that the data collectors were associated with a research project and developing a program that had been endorsed and encouraged by the local government and center leaders. The extent to which this resulted in social desirability biases among participants, who may have felt pressured to suppress negative assessments about technology, remains an open question. Finally, grounded theory research often involves cycles of analysis interspersed with data collection, allowing emergent themes to be explored in later interviews, however given the time and budgetary constraints of the project we were only able to conduct interviews in a single condensed timeframe.

## Conclusion

In conclusion, our study reveals that while the integration of *mHealth* campaigns may positively influence vaccine demand and streamline communication, it presents new challenges for CHWs. The expanding scope of the CHWs duties, coupled with the introduction of technology, highlight the need for balanced solutions that support CHWs without overwhelming them. In addition, our findings emphasize the need for *mHealth* solutions that enhance rather than replace the vital role of CHWs in fostering trust and overcoming barriers to childhood vaccination. Addressing vaccine hesitancy, particularly within religious contexts, requires tailored strategies that respect and understand these nuances. Optimizing *mHealth* tools to reduce administrative burdens and enhance their compatibility with existing systems is essential to truly maximize the benefits for CHWs and the communities they serve. Additionally, strengthening support mechanisms for CHWs will be crucial in maintaining their effectiveness and ensuring equitable access to healthcare services in underserved communities.

## Supporting information

S1 File (DOCX)
